# Leukocyte Subtype Counts and Its Association with Vascular Structure and Function in Adults with Intermediate Cardiovascular Risk. MARK Study

**DOI:** 10.1371/journal.pone.0119963

**Published:** 2015-04-17

**Authors:** Leticia Gomez-Sanchez, Luis García-Ortiz, José I. Recio-Rodríguez, Maria C. Patino-Alonso, Cristina Agudo-Conde, Fernando Rigo, Rafel Ramos, Ruth Martí, Manuel A. Gomez-Marcos, for the MARK Group

**Affiliations:** 1 Primary Care Research Unit, The Alamedilla Health Center, Salamanca, Spain; 2 Castilla and León Health Service (SACyL), Biomedical Research Institute of Salamanca (IBSAL), Salamanca, Spain; 3 Medicine Department, University of Salamanca, Salamanca, Spain; 4 Statistics Department, University of Salamanca, Salamanca, Spain; 5 San Agustín Health Center, Isles Baleares Health Service (IBSALUT), Palma de Mallorca, Spain; 6 Research Unit Family Medicine, Jordi Gol Institute for Primary Care Research (IDIAP Jordi Gol), Girona, Spain; 7 Translab Research Group, Department of Medical Sciences, School of Medicine, University of Girona, Girona, Spain; 8 Girona Biomedical Research Institute (IDIBGI), Dr. Trueta University Hospital, Girona, Spain; 9 MARK Group, Research Network on Preventive Activities and Health Promotion (redIAPP), Girona, Spain; University Hospital Medical Centre, GERMANY

## Abstract

**Objectives:**

We investigated the relationship between leukocyte subtype counts and vascular structure and function based on carotid intima-media thickness, pulse wave velocity, central augmentation index and cardio-ankle vascular index by gender in intermediate cardiovascular risk patients.

**Methods:**

This study analyzed 500 subjects who were included in the **MARK** study, aged 35 to 74 years (mean: 60.3±8.4), 45.6% women. Measurement: Brachial ankle Pulse Wave Velocity (ba-PWV) estimate by equation, Cardio-AnkleVascular Index (CAVI) using the VaSera device and Carotid ultrasound was used to measure carotid Intima Media Thickness (IMT). The Mobil-O-Graph was used to measure the Central Augmentation Index (CAIx).

**Results:**

Total leukocyte, neutrophil and monocyte counts were positively correlated with IMT (p < 0.01) in men. Monocyte count was positively correlated with CAIx in women (p < 0.01). In a multiple linear regression analysis, the IMT mean maintained a positive association with the neutrophil count (β = 1.500, p = 0.007) in men. CAIx maintained a positive association with the monocyte count (β = 2.445, p = 0.022) in women.

**Conclusion:**

The results of this study suggest that the relationship between subtype circulating leukocyte counts and vascular structure and function, although small, may be different by gender. In men, the neutrophil count was positively correlated with IMT and in women, the monocyte count with CAIx, in a large sample of intermediate-risk patients. These association were maintained after adjusting for age and other confounders.

**Trial Registration:**

ClinicalTrials.gov NCT01428934

## Introduction

Atherosclerosis is, at least in part, an inflammatory process involving the infiltration of the circulating leukocyte subtype into the vessel wall [1]. Leukocyte adhesion to the vascular endothelium and subsequent transendothelial migration play essential roles in the pathogenesis of cardiovascular diseases such as atherosclerosis [2]. Several epidemiologic studies have reported that an increased leukocyte subtype count is a strong independent risk factor for cardiovascular (CV) events [3,4]. An increased leukocyte subtype count is an independent risk factor for the prevalence and progression of subclinical carotid atherosclerosis [5–11]. However, Kuo et al. [12] reported no association between total leukocyte count and IMT in asymptomatic subjects.

The different parameters used to assess vascular structure and function are related to the risk of cardiovascular morbidity and mortality. Similarly, ultrasound examination of the carotid arteries with the measurement of intima media thickness (IMT) or the presence of plaques has been shown to predict the occurrence of both stroke and myocardial infarction, independently of traditional CV risk factors [13,14]. The pulse wave velocity (PWV) has been associated with increased morbidity-mortality in both patients with cardiovascular disease and in healthy individuals [15,16]. The central augmentation index (CAIx) is an independent predictor of all-cause and CV mortality [17]. The Cardio-Ankle vascular index (CAVI) is also used to evaluate arterial stiffness and can be used to estimate the risk of atherosclerosis. This parameter is independent of arterial pressure at measuring time [18,19].

The association between leukocyte subtype counts and vascular structure and function in patients with intermediate risk has not been determined.

Moreover, there are differences in the measures used to assess the arterial stiffness according to gender [20], and discrepancies in the behavior of different measures are currently not completely resolved [21].

In patients with intermediate risk, it is important to analyze new cardiovascular risk factors and the association between them for personalized risk stratification [22].

Currently, many studies that analyze new techniques or biomarkers to improve risk estimation based on scales have focused on the population with intermediate cardiovascular risk [3,23,24].

Therefore, we investigated the relationship between leukocyte subtype counts and vascular structure and function based on carotid intima-media thickness, pulse wave velocity, central augmentation index and cardio-ankle vascular index in intermediate risk patients.

## Methods

### Study Design

This is a cross-sectional study to evaluate if ankle-brachial index (ABI), measures of arterial stiffness (CAVI), postprandial glucose, glycosylated hemoglobin, self-measured blood pressure and presence of comorbidity are independently associated to incidence of vascular events and whether they can improve the predictive capacity of current risk equations in the intermediate risk population. The second step will be a following of 5 and 10 years to evaluate cardiovascular morbidity and mortality. This study analyzed 500 subjects who were included at the baseline of MARK study (NCT01428934) [25].

### Study Population

The population comprised individuals aged between 35 to 74 years who had intermediate cardiovascular risk, which was defined as coronary risk between 5% -15% at 10 years according to the Framingham adapted risk equation (REGICOR) [26] or vascular mortality risk between 2–5% at 10 years according to the SCORE equation [27]. The exclusion criteria were terminal illness, institutionalization at the appointment time, or a personal history of atherosclerotic disease. Sample selection was done with a random sample from the population aged 35 to 74 (both included) who had an intermediate cardiovascular risk. Recruitment and data collection for the study were carried out from July 2011to June 2013. A sample-size calculation indicated that the 500 patients included in the study constituted a sufficient sample for detecting a correlation coefficient of 0.125 between leukocyte subtype counts and IMT in a two sided test, with a level of significance of 95% (alfa risk 0.05) and a power of 80% (beta risk 0.20). We have used the EPIDAT 4.0 software to perform this estimation. The study was approved by an independent ethics committee of Salamanca University Hospital (Spain), and all participants gave written informed consent according to the general recommendations of the Declaration of Helsinki [28].

## Measurements

A detailed description has been published elsewhere regarding how the clinical data, anthropometric measurements, and analytical parameters were obtained [25].

### Laboratory determinations

Venous blood sampling was performed between 08:00 and 09:00 after the individuals had fasted and abstained from smoking and the consumption of alcohol and caffeinated beverages for the previous 12 hours. Fasting plasma glucose, serum total cholesterol and high-density lipoprotein (HDL) cholesterol concentrations were measured using standard enzymatic automated methods. Low-density lipoprotein (LDL) cholesterol was estimated by the Friedewald equation when the direct parameter was not available. Non-HDL cholesterol was estimated by the equation (Non-HDL Cholesterol = Total Cholesterol- HDL Cholesterol). Blood samples were collected in the Alamedilla Health Center and analyzed at the hospital of Salamanca, which was approved by the external quality assurance programs of the Spanish Society of Clinical Chemistry and Molecular Pathology.

### Office blood pressure

Office blood pressure (BP) was calculated as the average of the last two of three measurements of systolic blood pressure (SBP) and diastolic blood pressure (DBP) made with a validated sphygmomanometer (OMRON Model M10-IT). Measurements were made on the dominant arm of participants in the seated position after at least 5 minutes of rest, with a cuff of appropriate size as determined by measurement of the upper arm circumference and following the recommendations of the European Society of Hypertension [29].

### Central augmentation index (CAIx)

This parameter was estimated using an oscillometric Mobil-O-Graph (Stolberg, Germany) [30]. The device was validated according to British Hypertension Society [31] and European Society of Hypertension [32] recommendations. The measurements of central systolic blood pressure (cSBP) and peripheral systolic blood pressure (pSBP) were also taken on the dominant arm. Arm circumferences were measured and recorded to allow the correct choice of cuff size (two sizes available: 24–34 and 32–42 cm). With a conventional cuff, the determination of the cSBP is based on an oscillometric BP measurement and uses the pulse waves assessed at the brachial artery. After the estimation of peripheral BPs, the cuff instantly reinflates, and recordings for cSBP are carried out at diastolic pressure levels for 10 sec [33,34]. From the morphology of the aortic wave, CAIx was estimated using the following formula: increase in central pressure ×100/pulse pressure. The value was adjusted to a heart rate of 75 by the Mobil-O-Graph device.

### Cardio Ankle Vascular Index (CAVI) and brachial ankle Pulse Wave Velocity (ba-PWV)

CAVI was measured using a Vasera VS-1500 device (Fukuda Denshi) and ba-PWV estimated by validate equation. The ba-PWV and CAVI were calculated to give a more accurate calculation of the degree of atherosclerosis. CAVI integrates cardiovascular elasticity derived from the aorta to the ankle pulse velocity through an oscillometric method. Takaki et al. [35] provided the evidence for the validity of CAVI by showing a positive correlation between the stiffness parameter β of the aorta and CAVI (r = 0.67, p <0.01). The measurement of CAVI not affected by the increase in BP during measurement [18,19,36,37], CAVI values were automatically calculated by substituting the stiffness parameter β in the following equation to detect the vascular elasticity and the cardio-ankle PWV: Stiffness parameter β = 2ρ x 1/ (Ps-Pd) x ln (Ps/Pd) x PWV^2^, where ρ is the blood density, Ps and Pd are SBP and DBP in mmHg, and PWV is measured between the aortic valve and ankle. The average coefficient of variation of the CAVI is less than 5%, which is small enough for clinical use and confirms that CAVI has favorable reproducibility [18,38]. CAVI was measured at rest. CAVI was classified as normal (CAVI < 8), borderline (CAVI ≥ 8 and < 9) and abnormal (CAVI ≥ 9). Abnormal CAVI represents subclinical atherosclerosis. ba-PWV ≥ 17.5 m/sec was considered abnormal [39,40]. For the study, the higher obtained CAVI and ba-PWV were considered.

### Assessment of vascular structure by carotid intima media thickness (IMT)

Carotid ultrasound was performed to assess carotid IMT by two investigators trained for this purpose before starting the study. The reliability of the recordings was evaluated before the study using the intra-class correlation coefficient, which showed values of 0.97 (95% CI: 0.94 to 0.99) for intra-observer agreement in repeated measurements of 20 subjects, and 0.90 (95% CI: 0.74 to 0.96) for inter-observer agreement. According to the Bland-Altman analysis, the mean difference for inter-observer agreement (95% limits of agreement) was 0.01 (-0.03 to 0.06). A Sonosite Micromax ultrasound device paired with a 5–10 MHz multi-frequency high-resolution linear transducer with Sonocal software was used for performing automatic measurements of IMT in order to optimize reproducibility.

Measurements were made of the common carotid after the examination of a 10 mm longitudinal section at a distance of 1 cm from the bifurcation, performing measurements in the anterior and in the posterior wall, in the lateral, anterior and posterior projections, following an axis perpendicular to the artery to discriminate two lines: one for the intima-blood interface and the other for the media-adventitious interface. A total of 6 measurements of the right carotid were obtained, with another 6 measurements of the left carotid, using average values (average mean IMT and average maximum IMT) automatically calculated by the software [41]. The measurements were obtained with the subject lying down, with the head extended and slightly turned opposite to the examined carotid artery. Average mean IMT was considered abnormal if > 0.90 mm or if there were atherosclerotic plaques with a diameter of 1.5 mm or a focal increase of 0.5 mm or 50% of the adjacent IMT [42].

### Anthropometric measurements

Body weight was determined on two occasions using a homologated electronic scale (Seca 770) following calibration (precision ± 0.1 kg), with the patient wearing light clothing and no shoes. Height in turn was measured with a portable system (Seca 222), recording the average of two readings. Body mass index (BMI) was calculated as weight (kg) divided by height squared (m^2^). A value of > 30 kg/m^2^ was taken to define obesity. The individuals performing the different tests were blinded to the clinical data of the patient. All assessments were made within a period of 10 days.

### Statistical Analysis

Continuous variables were expressed as the mean ± standard deviation for normally distributed continuous data, the median (interquartile range, IQR) for asymmetrically distributed continuous data, and the frequency distribution for categorical data. Statistical normality was tested using the Kolmogorov–Smirnov test. A Spearman’s correlation was used to analyze the relationship between asymmetrically distributed continuous data. The difference of means between two categories of quantitative variables for asymmetrically distributed continuous data was analyzed using the Mann-Whitney U test. The difference of means between more than two categories of quantitative variables for symmetrically distributed continuous data was analyzed using the Anova test. We performed multiple linear regression analyses, one for each of the dependent variables, with IMT mean (x 100) and CAIx (x 10) to facilitate interpretation, ba-PWV and CAVI as dependent variables and total leukocyte count (/mm^3^), monocyte count (/mm^3^), neutrophil count (/mm^3^) and lymphocyte count (/mm^3^) as independent variables. We adjusted by age, systolic blood pressure, non-HDL cholesterol, fasting glucose, body mass index, number of cigarettes smoked, and antihypertensive, lipid-lowering and antidiabetic drugs. Comparisons between three or more groups were performed using ANOVA and differences between groups were assessed using DMS post hoc test. The data were analyzed using the Statistical Package for the Social Sciences version 20.0 (SPSS, Chicago, IL, USA). A value of p<0.05 was considered statistically significant.

## Results

The characteristics of the study subjects, global and by gender, are shown in Table 1. The mean age was 60.3 ± 8.4 years, and 45.6% of the subjects were women. IMT and PWV had higher values in men, and CAIx was higher in women (p<0.01). The total leukocyte, monocyte and neutrophil counts were higher in men.

**Table 1:** Baseline demographic and clinical characteristics of patients.

	Global n = 500	Women N = 228	Men N = 272	p-value
Age (years)	60.3±8.4	61.9±8.0	58.9±8.5	<0.001
Smoking n (%)	117 (23.4)	46 (39.3)	71(60.7)	0.138
Body mass index (kg/m^2^)	27.8 (25.3–30.3)	27.5 (24.9–30.7)	27.9 (25.9–30.1)	0.254
Obesity n (%)	137 (27.4)	68 (49.6)	69 (50.4)	0.270
Office SBP (mmHg)	133.9±16.6	130.9±17.0	136.3±15.9	<0.001
Office DBP (mmHg)	81.3±10.7	79.3±11.2	82.9±10.1	<0.001
Heart Rate (beats/min)	69 (63–77)	70 (65–78)	67 (61–76)	0.004
Hypertension n (%)	401 (80.2)	175(43.6)	226 (56.4)	0.091
Antihypertensive Drugs n (%)	266 (53.2)	120 (45.1)	146 (54.9)	0.857
Fasting glucose (mg/dL)	89 (82–99)	88 (82–98)	90 (82–100)	0.226
Diabetes n (%)	138 (27.6)	51 (37.0)	87 (63.0)	0.021
Antidiabetic drugs n (%)	82 (16.4)	30 (36.6)	52 (63.4)	0.089
Total cholesterol (mg/dL)	216.2±38.5	221.1±36.8	212.1±39.3	0.009
LDL-cholesterol (mg/dL)	135.1±34.3	136.1±33.4	134.9±35.2	0.850
HDL-cholesterol (mg/dL)	53.5 (44.7–63.9)	59.9 (51.2–69.5)	49.0 (41.5–56.9)	<0.001
Non-HDL cholesterol (mg/dL)	80.4±14.0	84.3±13.3	77.1±13.8	<0.001
Dyslipidemia n (%)	418 (83.6)	197 (47.1)	221 (52.9)	0.146
Lipid lowering drugs n (%)	185 (37.0)	86 (37.7)	99 (36.4)	0.781
Total leukocyte count *10^3^ (/mm^3^)	6.8 (5.7–8.1)	6.4 (5.4–7.6)	7.1 (6.1–8.3)	<0.001
Monocyte count *10^3^ (/mm^3^)	0.5 (0.5–0.7)	0.5 (0.4–0.6)	0.6 (0.5–0.7)	<0.001
Neutrophil count *10^3^ (/mm^3^)	3.5 (2.9–4.3)	3.3 (2.6–4.1)	3.7 (3.0–4.5)	<0.001
Lymphocyte count *10^3^ (/mm^3^)	2.4 (2.0–2.9)	2.4 (2.0–2.8)	2.4 (1.9–3.0)	0.472
IMT mean (mm)	0.74±0.09	0.72±0.08	0.75±0.10	<0.001
TOD carotid n (%)	85 (17.1)	86 (46.5)	99 (53.5)	0.781
CAIx (%)	26.78±13.80	31.39±13.72	22.68±12.66	<0.001
ba-PWV (m/sec)	14.16 (12.69–16.19)	14.74 (12.83–16.46)	13.86(12.55–15.99)	0.009
ba-PWV ≥ 17.5 n (%)	65 (13.1)	35 (53.8)	30 (46.2)	0.142
CAVI	8.59±1.10	8.55±1.03	8.63±1.16	0.449
CAVI ≥ 9 n (%)	155 (31.3)	46 (39.3)	71 (60.7)	0.138

Values are means (standard deviations (SD)) for normally distributed continuous data and medians (interquartile range (IQR)) for asymmetrically distributed continuous data and number and proportions for categorical data. SBP: Systolic Blood Pressure. DBP: Diastolic Blood Pressure. HDL: High Density Lipoprotein. Non HDL-Colesterol = Total Cholesterol—LDL-Cholesterol. LDL: Low Density Lipoprotein. IMT: Intima Media Thickness of common carotid artery. TOD: Target Organ Damage. CAIx: Central Augmentation Index corrected for heart rate. ba-PWV: Brachial Ankle Pulse Wave Velocity. CAVI: Cardio-Ankle Vascular Index.

Table 2 shows the values of the total leukocyte, monocyte, neutrophil and lymphocyte counts according to the target organ damage of IMT, ba-PWV and CAVI. There was a greater total leukocyte count and in all series in subjects with pathologic IMT.

**Table 2:** Leukocyte subtypes counts according parameters of vascular structure and function.

TOD carotid	With TOD	Without TOD	p value
Total leukocyte count *10^3^ (/mm^3^)	7.3 (6.2–8.6)	6.7 (5.6–7.9)	0.002
Monocyte count *10^3^ (/mm^3^)	0.6 (0.5–0.7)	0.5 (0.4–0.6)	0.006
Neutrophil count *10^3^ (/mm^3^)	3.7 (3.1–4.9)	3.5 (2.9–4.3)	0.005
Lymphocyte count (/mm^3^)	2.6 (2.1–3.2)	2.4 (2.0–2.9)	0.059
**ba-PWV ≥ 17.5 m/sec**			
Total leukocyte count *10^3^ (/mm^3^)	6.9 (5.9–7.7)	6.7 (5.6–8.1)	0.711
Monocyte count *10^3^ (/mm^3^)	0.5 (0.4–0.6)	0.5 (0.5–0.7)	0.700
Neutrophil count*10^3^(/mm^3^)	3.6 (2.9–4.4)	3.5 (2.9–4.3)	0.881
Lymphocyte count*10^3^(/mm^3^)	2.5 (2.1–3.2)	2.4 (2.0–2.9)	0.219
**CAVI ≥ 9**			
Total leukocyte count *10^3^ (/mm^3^)	6.7 (5.6–7.7)	6.8 (5.7–8.3)	0.291
Monocyte count *10^3^ (/mm^3^)	0.5 (0.4–0.6)	0.5 (0.5–0.7)	0.922
Neutrophil count*10^3^(/mm^3^)	3.4 (2.9–4.2)	3.6 (2.9–4.4)	0.188
Lymphocyte count*10^3^(/mm^3^)	2.4 (2.0–2.9)	2.4 (2.0–2.9)	0.993

Values are medians (interquartile range (IQR)). TOD: Target Organ Damage. ba-PWV: Brachial Ankle Pulse Wave Velocity. CAVI: Cardio-Ankle Vascular Index. TOD carotid: Intima Media Thickness (IMT) > 0.90 mm or plaques with a diameter of 1.5 mm or a focal increase of 0.5 mm or 50% of the adjacent IMT

**Fig 1:**
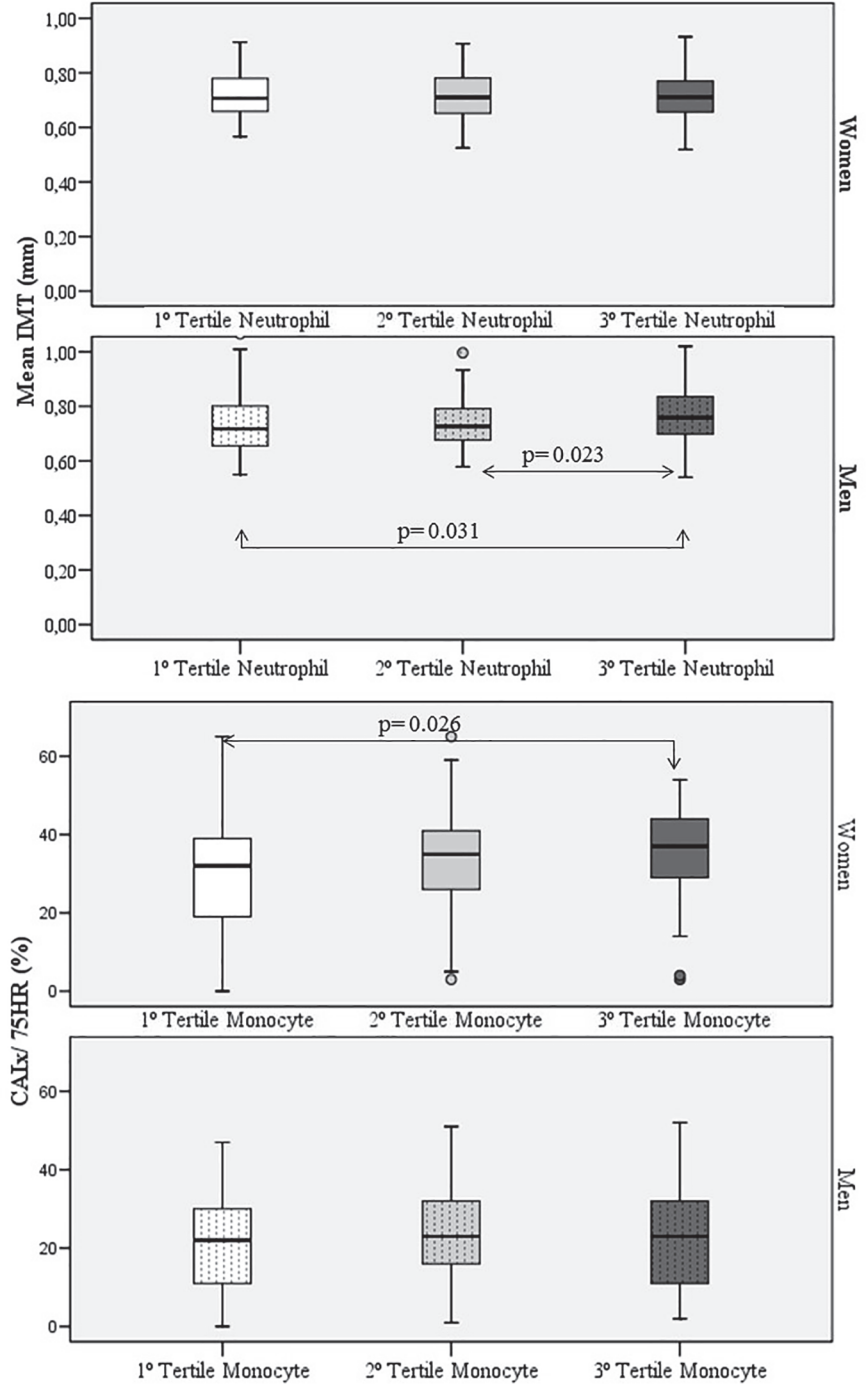
Mean IMT values according to the neutrophil count tertiles in men; Neutrophil count tertiles: T1 < 3000/mm^3^; T2 3000 to 4000/mm^3^; T3 > 4000/mm^3^. CAIx in women values according to the monocyte count tertiles. Monocyte/count tertiles: 1° Tertile < 500 /mm^3^, 2° Tertile 500 to 600/mm^3^, 3° Tertile > 600 /mm^3^. ANOVA Test: Carotid intima media thickness by neutrophil count tertiles in men: p = 0.035; CAIx by monocyte count tertiles in women: p = 0.042.

Fig. 1 shows the values of the IMT by neutrophil count tertiles and the CAIx by monocyte count tertiles.

**Fig 2:**
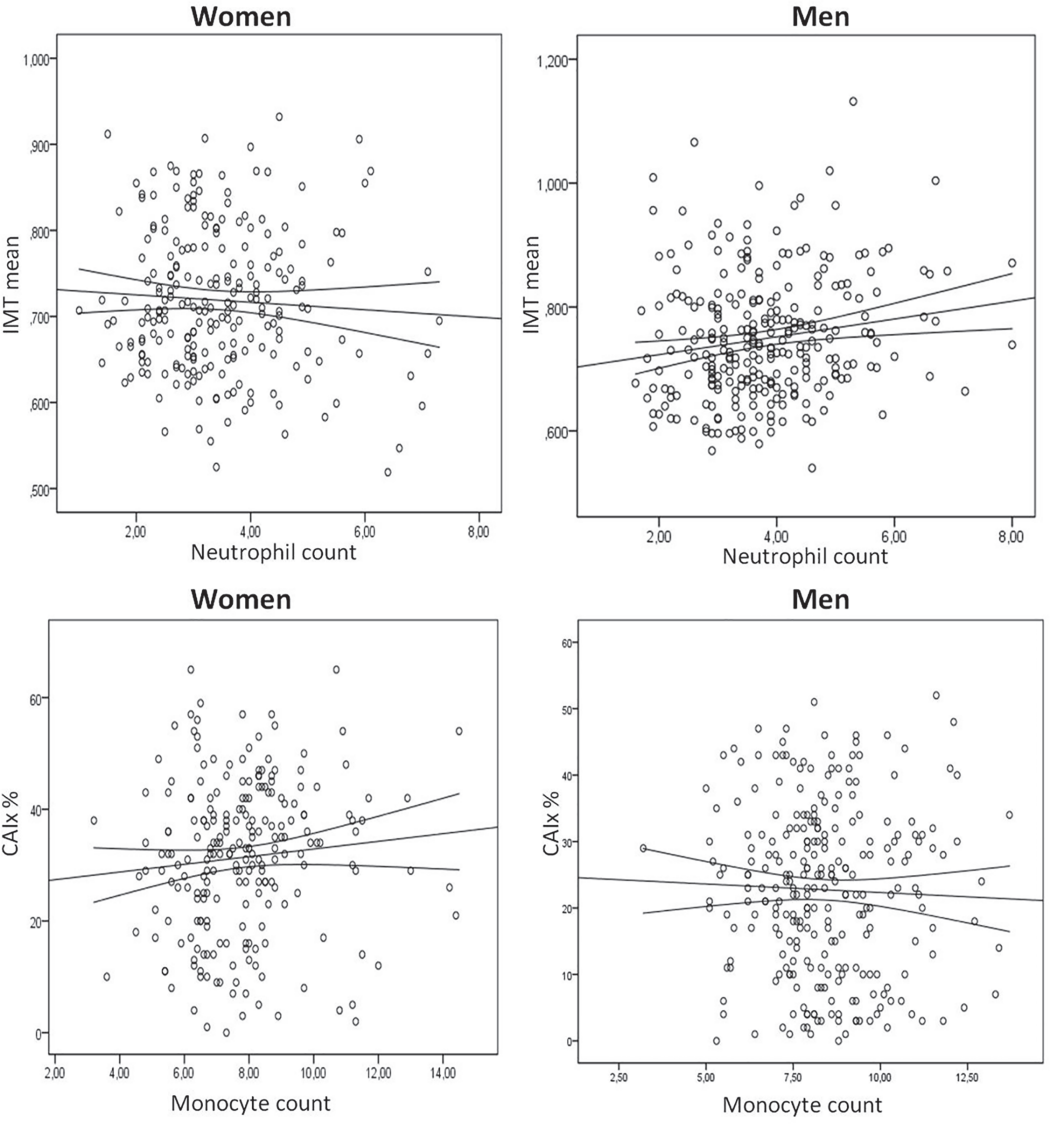
IMT regression line with Neutrophil count: Women: (r = -0.019, p = 0.781); Men: (r = 0.170, p = 0.005); CAIx regression line with monocyte count: Women: (r = 0.212, p = 0.001); Men: r = 0.030, p = 0.629).

The total leukocyte, neutrophil and monocyte counts were positively correlated with mean average IMT (p<0.01) in men. The monocyte count was positively correlated with CAIx in women (r = 0.212; p<0.01) (Table 3). Simple linear regressions in Fig. 2.

**Table 3:** Bivariate correlations of vascular structure and function indices with leukocyte subtype counts.

	Total leukocyte count (/mm^3^)	Monocyte count (/mm^3^)	Neutrophil count (/mm^3^)	Lymphocyte count (/mm^3^)
**IMT mean:**				
Global	**0.104** *	**0.128** **	**0.103** *	**0.089** *
Men	**0.167** **	**0.152** *	**0.170** **	0.093
Women	−0.010	0.015	−0.019	0.086
**CAIx**				
Global	0.027	0.017	−0.007	0.076
Men	0.062	0.030	0.037	0.070
Women	0.116	**0.212** **	0.048	0.127
**ba-PWV:**				
Global	0.039	−0.019	0.045	0.069
Men	0.081	0.067	0.078	0.086
Women	0.035	−0.053	0.055	0.056
**CAVI**				
Global	−0.049	−0.012	−0.058	0.014
Men	−0.056	0.012	−0.053	−0.019
Women	−0.041	−0.050	−0.067	0.052

IMT: Intima Media Thickness of common carotid artery. CAIx: Central Augmentation Index corrected for heart rate. ba-PWV: Brachial Ankle Pulse Wave Velocity. CAVI: Cardio-Ankle Vascular Index.

p-values by Spearman correlation.

* p<0.05

** p<0.01.

A multiple linear regression analysis by gender was carried out (Table 4). After adjusting for age, systolic blood pressure, non-HDL cholesterol, fasting glucose, body mass index, number of cigarettes smoked, antihypertensive drugs, lipid-lowering drugs and antidiabetic drugs, the mean IMT maintained a positive association with the neutrophil count (β = 1.500; p = 0.007) in men. CAIx maintained a positive association with the monocyte count (β = 2.445; p = 0.022) in women.

**Table 4:** Multiple Regression Analysis with vascular structure and function parameters as dependent variable and leukocyte subtype counts as independent variable.

Dependent variable:	Men			Women		
	β	CI 95%	p value	β	CI 95%	p value
IMT mean						
**Total leukocyte count (/mm3)**	**0.721**	**0.002 to1.439**	**0.049**	0.234	-0.590 to 1.058	0.574
Monocyte count (/mm^3^)	2.281	-4.713to 9.274	0.521	7.007	-3.887 to 17.901	0.204
**Neutrophil count (/mm** ^**3**^)	**1.500**	**0.409 to 2.592**	**0.007**	0.247	-1.049 to 1.543	0.705
Lymphocyte count (/mm^3^)	0.225	-1.369 to 1.818	0.781	0.651	-1.942 to 3.243	0.619
**CAIx**						
Total leukocyte count (/mm3)	0.081	-0.023to 0.185	0.128	0.028	-0.133 to 0.190	0.726
**Monocyte count (/mm3)**	0.225	-0.785to 1.237	0.660	**2.445**	**0.359 to 4.530**	**0.022**
Neutrophil count (/mm3)	0.076	-0.085to 0.237	0.355	-0.014	-0.268 to 0.240	0,912
Lymphocyte count (/mm3)	0.223	-0.004to 0.450	0.055	-0.029	-0.539 to 0.479	0.908
**ba-PWV**						
Total leukocyte count (/mm3)	0.130	-0.082to 0.342	0.229	-0.016	-0.213 to 0.180	0.869
Monocyte count (/mm3)	0.955	-1.091to 3.002	0.358	-1.346	-3.950 to 1.257	0.306
Neutrophil count (/mm3)	0.169	-0.156to 0.494	0.308	-0.002	-0.310 to 0.307	0.991
Lymphocyte count (/mm3)	0.288	-0.176to 0.753	0.222	0.352	-0.261 to 0.964	0.257
**CAVI**						
Total leukocyte count (/mm3)	0.025	-0.050to 0.101	0.508	-0.047	-0.143 to 0.49	0.335
Monocyte count (/mm3)	0.036	-0.693to 0.765	0.922	-1.009	-2.276 to 0.257	0.117
Neutrophil count (/mm3)	0.019	-0.097to 0.135	0.745	-0.060	-0.211 to 0.091	0.428
Lymphocyte count (/mm3)	0.103	-0.063to 0.268	0.222	0.143	-0.159 to 0.444	0.349

**Dependent variables:** IMT mean: Intima Media thickness of common carotid artery. CAIx: Central Augmentation Index corrected for heart rate. ba-PWV: Brachial ankle Pulse Wave Velocity. CAVI: Cardio-Ankle Vascular Index. **Indepedent variables:** Total leukocyte count. Monocyte count. Neutrophil count. Lymphocyte count. **Adjusted by:** Age, systolic blood pressure, Non-high density lipoprotein, fasting glucose, body mass index, number of cigarettes, antihypertensive drugs, lipid lowering drugs and antidiabetic drugs.

## Discussion

The results of this study suggest that the relationship between subtype circulating leukocyte counts and vascular structure and function, although small, may be different by gender. The monocyte count was positively association with CAIx in women, and the neutrophil count was with IMT in men in a large sample of intermediate cardiovascular risk patients.

Several studies have demonstrated that neutrophils have an important role in the atherosclerosis development in the general population [10,43] and in men [7]. The monocytes are correlated with the IMT in healthy subjects [5,11] and in Japanese subjects with type 2 diabetes [4].

The results of this study are consistent with those previously disclosed and add new information. According to our results, this correlation is observed only in men, and suggest that the role of neutrophils may be more important than monocytes when IMT is increased in men with mid-level cardiovascular risk. Our results support the paper published by Li et al. [44], who found a relation only in men between a high number of leucocytes and a high incidence rate of cardio vascular diseases, but not with stroke. In summary, the leukocytes seem to have an independent role in early arterial damage and they may reflect subclinical disease in men.

As far as we know, this is the first study that describes a positive association between monocytes and CAIx in women. The assessment of CAIx is a simple approach to quantify the role of wave reflection in determining an elevation of central blood pressure values [16,45]. This relationship between CAIx and monocytes in women opens new lines of research.

The differences found between the two genders may be due to several factors. First, IMT is higher in men, while CAIx is higher in women. The influence of sex steroids on vascular function has been demonstrated in association with hormonal changes throughout the lifespan (puberty in both genders and menopause in females) [20,46]. Furthermore, anthropometric factors such as distribution of body fat [47], height, and aortic length are different between men and women [20,48,49]. Finally, the role of inflammatory components in the development of atherosclerosis is likely to differ according to gender. Thus, in a study conducted by our group in hypertensive patients, we found that hs-CRP has a positive association with IMT in men and a negative association with CAIx in women [50]. This result suggests that gender differences in cardiovascular outcomes may be mediated in part via gender differences in artery wall properties throughout life and that further understanding may lead to improved strategies for the prevention of cardiovascular diseases in both women and men.

The relation between the ba-PWV with the leukocyte subtype counts is not clear. In the same line as our results, Kavousiet al. [4] found that ba-PWV was not correlated with monocyte count, whereas Phillips et al. [11] found that leukocyte count (β = 0.07; p < 0.001) and granulocyte number (β = 0.07; p < 0.001) were significantly positively related to ba-PWV in a Chinese population, but not lymphocyte number. Bearing in mind these opposite results, it is considered as necessary to carry out more prospective studies in order to elucidate the association between ba-PWV and leukocyte subtype counts and the differences that could be observed in each gender.

The only study that has been found that analyzed the relationship between subtype circulating leukocyte counts with CAVI in 3738 Japanese people in the general population showed that the leukocyte counts weakly correlated with CAVI in men (β = 0.61; p = 0.043), but not in women (β = 0.35; p = 0.17) [51]. Our study has not shown any correlation between the leukocyte counts or their subtypes with the CAVI in neither men nor women.

### Limitations

This study has several limitations what should be considered in the interpretation of our results. Because this was a cross-sectional, observational study, which prevented us from establishing a temporal relationship between the circulating leukocyte subtype counts and the different parameters used to assess vascular function and structure and not allow us to establish a causal relationship. Some subjects were on treatment with antihypertensive or lipid lowering drugs, which might have affected the arterial stiffness measures. However, we adjusted for antihypertensive, lipid lowering and antidiabetic drugs. Finally, the correlations were small and need to be confirmed in longitudinal studios the clinical importance of them. However, this is the first study to examine the relationship between the circulating leukocyte subtype counts and different vascular structure and function parameters in intermediate-risk patients.

## Conclusions

The results of this study suggest that the relationship between subtype circulating leukocyte counts and vascular structure and function, although small, may be different by gender. In men, the neutrophil count was positively correlated with IMT and in women, the monocyte count with CAIx, in a large sample of intermediate-risk patients. These association were maintained after adjusting for age and other confounders.

Further research involving prospective studies are required to define the role of these leukocyte subtype counts in the vascular structure and function. In addition, possible gender-specific roles of leukocyte subtype counts remain to be elucidated.

## Supporting Information

S1 Data (SAV)

## References

[1] Tuttolomondo A, Di Raimondo D, Pecoraro R, Arnao V, Pinto A, Licata G: Atherosclerosis as an inflammatory disease. Curr Pharm Des 2012;18:4266–4288. 22390643 10.2174/138161212802481237

[2] Zhang J, Alcaide P, Liu L, Sun J, He A, Luscinskas FW, et al: Regulation of endothelial cell adhesion molecule expression by mast cells, macrophages, and neutrophils. PLoS One 2011;6:e14525 10.1371/journal.pone.0014525 21264293 PMC3021513

[3] Kavousi M, Elias-Smale S, Rutten JH, Leening MJ, Vliegenthart R, Verwoert GC, et al: Evaluation of newer risk markers for coronary heart disease risk classification: a cohort study. Ann Intern Med 2012;156:438–444. 10.7326/0003-4819-156-6-201203200-00006 22431676

[4] Matsumura T, Taketa K, Motoshima H, Senokuchi T, Ishii N, Kinoshita H, et al: Association between circulating leukocyte subtype counts and carotid intima-media thickness in Japanese subjects with type 2 diabetes. Cardiovasc Diabetol 2013;12:177 10.1186/1475-2840-12-177 24373412 PMC3878795

[5] Chapman CM, Beilby JP, McQuillan BM, Thompson PL, Hung J: Monocyte count, but not C-reactive protein or interleukin-6, is an independent risk marker for subclinical carotid atherosclerosis. Stroke 2004;35:1619–1624. 15155967 10.1161/01.STR.0000130857.19423.ad

[6] Johnsen SH, Fosse E, Joakimsen O, Mathiesen EB, Stensland-Bugge E, Njolstad I, et al: Monocyte count is a predictor of novel plaque formation: a 7-year follow-up study of 2610 persons without carotid plaque at baseline the Tromso Study. Stroke 2005;36:715–719. 15746459 10.1161/01.STR.0000158909.07634.83

[7] Loimaala A, Rontu R, Vuori I, Mercuri M, Lehtimaki T, Nenonen A, et al: Blood leukocyte count is a risk factor for intima-media thickening and subclinical carotid atherosclerosis in middle-aged men. Atherosclerosis 2006;188:363–369. 16378612 10.1016/j.atherosclerosis.2005.11.021

[8] Nozawa N, Hibi K, Endo M, Sugano T, Ebina T, Kosuge M, et al: Association between circulating monocytes and coronary plaque progression in patients with acute myocardial infarction. Circ J 2010;74:1384–1391. 20467155 10.1253/circj.cj-09-0779

[9] Lau KK, Wong YK, Chan YH, Yiu KH, Teo KC, Li LS, et al: Prognostic implications of surrogate markers of atherosclerosis in low to intermediate risk patients with type 2 diabetes. Cardiovasc Diabetol 2012;11:101 10.1186/1475-2840-11-101 22900680 PMC3444371

[10] Ortega E, Gilabert R, Nunez I, Cofan M, Sala-Vila A, de Groot E, et al: White blood cell count is associated with carotid and femoral atherosclerosis. Atherosclerosis 2012;221:275–281. 10.1016/j.atherosclerosis.2011.12.038 22244768

[11] Phillips AC, Jiang CQ, Thomas GN, Lin JM, Yue XJ, Cheng KK, et al: White blood cell subsets are associated with carotid intima-media thickness and pulse wave velocity in an older Chinese population: the Guangzhou Biobank Cohort Study. J Hum Hypertens 2012;26:485–492. 10.1038/jhh.2011.58 21654852

[12] Kuo WK, Lee SY, Ma SM, Ling TA, Wu CC: Correlation of hematologic factors to carotid intima-media thickness in men and women: a study of 2767 asymptomatic subjects of Taiwan. Acta Neurol Taiwan 2012;21:158–164. 23329546

[13] O'Leary DH, Polak JF, Kronmal RA, Manolio TA, Burke GL, Wolfson SK Jr.: Carotid-artery intima and media thickness as a risk factor for myocardial infarction and stroke in older adults. Cardiovascular Health Study Collaborative Research Group. N Engl J Med 1999;340:14–22. 9878640 10.1056/NEJM199901073400103

[14] Nambi V, Chambless L, Folsom AR, He M, Hu Y, Mosley T, et al: Carotid intima-media thickness and presence or absence of plaque improves prediction of coronary heart disease risk: the ARIC (Atherosclerosis Risk In Communities) study. J Am Coll Cardiol 2010;55:1600–1607. 10.1016/j.jacc.2009.11.075 20378078 PMC2862308

[15] Mattace-Raso FU, van der Cammen TJ, Hofman A, van Popele NM, Bos ML, Schalekamp MA, et al: Arterial stiffness and risk of coronary heart disease and stroke: the Rotterdam Study. Circulation 2006;113:657–663. 16461838 10.1161/CIRCULATIONAHA.105.555235

[16] Laurent S, Cockcroft J, Van Bortel L, Boutouyrie P, Giannattasio C, Hayoz D, et al: Expert consensus document on arterial stiffness: methodological issues and clinical applications. Eur Heart J 2006;27:2588–2605. 17000623 10.1093/eurheartj/ehl254

[17] Vlachopoulos C, Aznaouridis K, Stefanadis C: Prediction of cardiovascular events and all-cause mortality with arterial stiffness: a systematic review and meta-analysis. J Am Coll Cardiol 2010;55:1318–1327. 10.1016/j.jacc.2009.10.061 20338492

[18] Shirai K, Utino J, Otsuka K, Takata M: A novel blood pressure-independent arterial wall stiffness parameter; cardio-ankle vascular index (CAVI). J Atheroscler Thromb 2006;13:101–107. 16733298 10.5551/jat.13.101

[19] Shirai K, Hiruta N, Song M, Kurosu T, Suzuki J, Tomaru T, et al: Cardio-ankle vascular index (CAVI) as a novel indicator of arterial stiffness: theory, evidence and perspectives. J Atheroscler Thromb 2011;18:924–938. 21628839 10.5551/jat.7716

[20] Rossi P, Frances Y, Kingwell BA, Ahimastos AA: Gender differences in artery wall biomechanical properties throughout life. J Hypertens 2011;29:1023–1033. 10.1097/HJH.0b013e328344da5e 21346620

[21] Vermeersch SJ, Rietzschel ER, De Buyzere ML, De Bacquer D, De Backer G, Van Bortel LM, et al: Age and gender related patterns in carotid-femoral PWV and carotid and femoral stiffness in a large healthy, middle-aged population. J Hypertens 2008;26:1411–1419. 10.1097/HJH.0b013e3282ffac00 18551018

[22] Zhu J, Chen T, Yang L, Li Z, Wong MM, Zheng X, et al: Regulation of microRNA-155 in atherosclerotic inflammatory responses by targeting MAP3K10. PLoS One 2012;7:e46551 10.1371/journal.pone.0046551 23189122 PMC3506618

[23] Greenland P, Alpert JS, Beller GA, Benjamin EJ, Budoff MJ, Fayad ZA, et al: 2010 ACCF/AHA guideline for assessment of cardiovascular risk in asymptomatic adults: a report of the American College of Cardiology Foundation/American Heart Association Task Force on Practice Guidelines. Circulation 2010;122:e584–636. 10.1161/CIR.0b013e3182051b4c 21098428

[24] Yeboah J, McClelland RL, Polonsky TS, Burke GL, Sibley CT, O'Leary D, et al: Comparison of novel risk markers for improvement in cardiovascular risk assessment in intermediate-risk individuals. JAMA 2012;308:788–795. 10.1001/jama.2012.9624 22910756 PMC4141475

[25] Marti R, Parramon D, Garcia-Ortiz L, Rigo F, Gomez-Marcos MA, Sempere I, et al: Improving interMediAte risk management. MARK study. BMC Cardiovasc Disord 2011;11:61 10.1186/1471-2261-11-61 21992621 PMC3207912

[26] Marrugat J, D'Agostino R, Sullivan L, Elosua R, Wilson P, Ordovas J, et al: An adaptation of the Framingham coronary heart disease risk function to European Mediterranean areas. J Epidemiol Community Health 2003;57:634–638. 12883073 10.1136/jech.57.8.634 PMC1732543

[27] Conroy RM, Pyorala K, Fitzgerald AP, Sans S, Menotti A, De Backer G, et al: Estimation of ten-year risk of fatal cardiovascular disease in Europe: the SCORE project. Eur Heart J 2003;24:987–1003. 12788299 10.1016/s0195-668x(03)00114-3

[28] World Medical Association Declaration of Helsinki: ethical principles for medical research involving human subjects. JAMA 2013;310:2191–2194. 10.1001/jama.2013.281053 24141714

[29] O'Brien E, Asmar R, Beilin L, Imai Y, Mancia G, Mengden T, et al: Practice guidelines of the European Society of Hypertension for clinic, ambulatory and self blood pressure measurement. J Hypertens 2005;23:697–701. 15775768 10.1097/01.hjh.0000163132.84890.c4

[30] Wassertheurer S, Kropf J, Weber T, van der Giet M, Baulmann J, Ammer M, et al: A new oscillometric method for pulse wave analysis: comparison with a common tonometric method. J Hum Hypertens 2010;24:498–504. 10.1038/jhh.2010.27 20237499 PMC2907506

[31] Wei W, Tolle M, Zidek W, van der Giet M: Validation of the mobil-O-Graph: 24 h-blood pressure measurement device. Blood Press Monit 2010;15:225–228. 10.1097/MBP.0b013e328338892f 20216407

[32] Franssen PM, Imholz BP: Evaluation of the Mobil-O-Graph new generation ABPM device using the ESH criteria. Blood Press Monit 2010;15:229–231. 20658764 10.1097/mbp.0b013e328339be38

[33] Protogerou AD, Argyris A, Nasothimiou E, Vrachatis D, Papaioannou TG, Tzamouranis D, et al: Feasibility and reproducibility of noninvasive 24-h ambulatory aortic blood pressure monitoring with a brachial cuff-based oscillometric device. Am J Hypertens 2012;25:876–882. 10.1038/ajh.2012.63 22673021

[34] Weiss W, Gohlisch C, Harsch-Gladisch C, Tolle M, Zidek W, van der Giet M: Oscillometric estimation of central blood pressure: validation of the Mobil-O-Graph in comparison with the SphygmoCor device. Blood Press Monit 2012;17:128–131. 10.1097/MBP.0b013e328353ff63 22561735

[35] Takaki A, Ogawa H, Wakeyama T, Iwami T, Kimura M, Hadano Y, et al: Cardio-ankle vascular index is a new noninvasive parameter of arterial stiffness. Circ J 2007;71:1710–1714. 17965489 10.1253/circj.71.1710

[36] Shirai K, Song M, Suzuki J, Kurosu T, Oyama T, Nagayama D, et al: Contradictory effects of beta1- and alpha1- aderenergic receptor blockers on cardio-ankle vascular stiffness index (CAVI)—CAVI independent of blood pressure. J Atheroscler Thromb 2011;18:49–55. 21071883 10.5551/jat.3582

[37] Takaki A, Ogawa H, Wakeyama T, Iwami T, Kimura M, Hadano Y, et al: Cardio-ankle vascular index is superior to brachial-ankle pulse wave velocity as an index of arterial stiffness. Hypertens Res 2008;31:1347–1355. 10.1291/hypres.31.1347 18957805

[38] Kubozono T, Miyata M, Ueyama K, Nagaki A, Otsuji Y, Kusano K, et al: Clinical significance and reproducibility of new arterial distensibility index. Circ J 2007;71:89–94. 17186984 10.1253/circj.71.89

[39] Korkmaz L, Erkan H, Korkmaz AA, Acar Z, Agac MT, Bektas H, et al: Relationship of aortic knob width with cardio-ankle vascular stiffness index and its value in diagnosis of subclinical atherosclerosis in hypertensive patients: a study on diagnostic accuracy. Anadolu Kardiyol Derg 2012;12:102–106. 10.5152/akd.2012.034 22281788

[40] Kawai T, Ohishi M, Onishi M, Ito N, Takeya Y, Maekawa Y, et al: Cut-off value of brachial-ankle pulse wave velocity to predict cardiovascular disease in hypertensive patients: a cohort study. J Atheroscler Thromb 2013;20:391–400. 23268984 10.5551/jat.15040

[41] Gomez-Marcos MA, Recio-Rodriguez JI, Patino-Alonso MC, Agudo-Conde C, Gomez-Sanchez L, Gomez-Sanchez M, et al: Protocol for measuring carotid intima-media thickness that best correlates with cardiovascular risk and target organ damage. Am J Hypertens 2012;25:955–961. 10.1038/ajh.2012.72 22717546

[42] Mancia G, Fagard R, Narkiewicz K, Redon J, Zanchetti A, Bohm M, et al: 2013 ESH/ESC Guidelines for the management of arterial hypertension: the Task Force for the management of arterial hypertension of the European Society of Hypertension (ESH) and of the European Society of Cardiology (ESC). J Hypertens 2013;31:1281–1357. 10.1097/01.hjh.0000431740.32696.cc 23817082

[43] Soehnlein O: Multiple roles for neutrophils in atherosclerosis. Circ Res 2012;110:875–888. 10.1161/CIRCRESAHA.111.257535 22427325

[44] Li C, Engstrom G, Hedblad B: Leukocyte count is associated with incidence of coronary events, but not with stroke: a prospective cohort study. Atherosclerosis 2010;209:545–550. 10.1016/j.atherosclerosis.2009.09.029 19833340

[45] McEniery CM, Yasmin, Maki-Petaja KM, McDonnell BJ, Munnery M, Hickson SS, et al: The impact of cardiovascular risk factors on aortic stiffness and wave reflections depends on age: the Anglo-Cardiff Collaborative Trial (ACCT III). Hypertension 2010;56:591–597. 10.1161/HYPERTENSIONAHA.110.156950 20696989

[46] Marlatt KL, Kelly AS, Steinberger J, Dengel DR: The influence of gender on carotid artery compliance and distensibility in children and adults. J Clin Ultrasound 2013;41:340–346. 10.1002/jcu.22015 23233368 PMC3736987

[47] Anoop S, Misra A, Bhardwaj S, Gulati S: High body fat and low muscle mass are associated with increased arterial stiffness in Asian Indians in North India. J Diabetes Complications 2014. 10.1016/j.jdiacomp.2014.08.001 25200813

[48] McEniery CM, Yasmin, Hall IR, Qasem A, Wilkinson IB, Cockcroft JR: Normal vascular aging: differential effects on wave reflection and aortic pulse wave velocity: the Anglo-Cardiff Collaborative Trial (ACCT). J Am Coll Cardiol 2005;46:1753–1760. 16256881 10.1016/j.jacc.2005.07.037

[49] Yasmin, Brown MJ: Similarities and differences between augmentation index and pulse wave velocity in the assessment of arterial stiffness. QJM 1999;92:595–600. 10627881 10.1093/qjmed/92.10.595

[50] Gomez-Marcos MA, Recio-Rodriguez JI, Patino-Alonso MC, Agudo-Conde C, Gomez-Sanchez L, Rodriguez-Sanchez E, et al: Relationships between high-sensitive C-reactive protein and markers of arterial stiffness in hypertensive patients. Differences by sex. BMC Cardiovasc Disord 2012;12:37 10.1186/1471-2261-12-37 22676422 PMC3473264

[51] Sekitani Y, Hayashida N, Kadota K, Yamasaki H, Abiru N, Nakazato M, et al: White blood cell count and cardiovascular biomarkers of atherosclerosis. Biomarkers 2010;15:454–460. 10.3109/1354750X.2010.486870 20507253

